# Pharmaceutical Contaminants Occurrence and Ecological Risk Assessment Along the Romanian Black Sea Coast

**DOI:** 10.3390/toxics13060498

**Published:** 2025-06-13

**Authors:** Vasile-Ion Iancu, Laura-Florentina Chiriac, Iuliana Paun, Cristina Dinu, Florinela Pirvu, Victor Cojocaru, Anda Gabriela Tenea, Ioana Antonia Cimpean

**Affiliations:** National Research and Development Institute for Industrial Ecology—ECOIND, Drumul Podu Dambovitei Street, 57–73, Sector 6, 060652 Bucharest, Romania; laura.chiriac@incdecoind.ro (L.-F.C.); iuliana.paun@incdecoind.ro (I.P.); florinela.pirvu@incdecoind.ro (F.P.); victor.cojocaru@ecoind.ro (V.C.); anda.tenea@incdecoind.ro (A.G.T.); antonia.cimpean@ecoind.ro (I.A.C.)

**Keywords:** NSAIDs, seawater, solid-phase extraction—SPE, liquid chromatography mass spectrometry in tandem—LC-MS/MS, ecological risk assessment

## Abstract

The work aimed to investigate the presence of pharmaceutical compounds from the anti-inflammatory class in seawater from the Romanian Black Sea coast and to assess the ecological risk of these substances on the most sensitive organisms. Using the solid-phase extraction technique (SPE) followed by liquid chromatography separation and mass spectrometry detection (LC-MS/MS) of the compounds, the concentrations of these contaminants in selected seawater samples were determined. Ibuprofen was the most commonly detected compound with a frequency of 42.9%, followed by ketoprofen at 31.0.%, diclofenac at 23.8%, and naproxen at 21.4%. The maximum concentrations of pharmaceutical products varied between 13.4 ng/L ketoprofen and 13,575 ng/L caffeine. The order of decreasing maximum concentrations of pharmaceutical compounds in the water of the Black Sea was CAF > IBU > NAP > DIC > KET. The dominant and ubiquitous compound that was determined with the maximum concentration values was caffeine. Strong correlations were observed between three compounds (naproxen: diclofenac, diclofenac: ketoprofen) suggesting the same pollution source. Through the ecological risk assessment, it was observed that both caffeine and ibuprofen can generate high ecological risks for some echinoderms, crustaceans, and fish.

## 1. Introduction

In the last decade, the scientific world has paid special attention to pharmaceutical compounds present in the aquatic environment due to the negative toxicological effects of these molecules on aquatic organisms and their persistence in rivers. The presence of drugs in the marine environment is little studied, although it is the last receptor of continental contamination. There is a need to develop new methods for analyzing emerging contaminants in coastal waters and to assess the ecological risks these compounds pose to the most sensitive marine organisms [1].

Pharmaceutically active compounds constitute a significant group of emerging contaminants in the environment [2]. Pharmaceuticals enter the aquatic environment primarily through human and animal excretion, improper disposal of unused medications, and agricultural and zootechnical practices [3]. The discharge of both treated and untreated wastewater represents the primary pathway through which pharmaceuticals and other contaminants enter the aquatic environment [4,5]. Due to their continuous release into the environment, pharmaceuticals are classified as persistent chemicals capable of exerting toxic effects on aquatic organisms [6,7]. More than 4000 pharmaceutical products are used globally for human medicine, veterinary healthcare, and to promote animal husbandry [1]. Drugs are compounds designed to interact with specific physiological pathways in target organisms. However, when they enter the marine environment, they can pose ecological risks to marine life. These substances may act as additional stressors on marine ecosystems that are already under pressure from climate change, overfishing, and eutrophication [8,9]. Adverse ecological effects of various pharmaceuticals can occur even at sub-lethal concentrations, altering biochemical and cellular responses in aquatic organisms. Essential biological functions—such as reproduction, growth, metabolism, immunity, feeding, and locomotion—may be disrupted depending on the type of drug and its specific pharmacological properties [10].

After administration, pharmaceuticals are excreted as a mixture of parent compounds and metabolites, which are often more polar and hydrophilic than the original drug. A portion of these substances enters wastewater as degradation products, many of which are poorly removed by conventional wastewater treatment plants [11,12]. The presence of pharmaceutical compounds in the environment is an increasing global concern, highlighting the need for environmental risk assessments that consider new classes of aquatic organisms sensitive to the potentially toxic effects of these substances. Pharmaceuticals predominantly enter the aquatic environment and are typically detected at concentrations ranging from sub-nanograms per liter (ng/L) to several micrograms per liter (μg/L) [13]. In the European Union, studies on the aquatic effects of pharmaceutical compounds are initiated when environmental concentrations exceed 0.01 μg/L [14,15]. Additionally, effect studies are mandated for drugs that are highly lipophilic (log D ≥ 4.50) or are potential endocrine disruptors, as they may interfere with reproductive functions. The occurrence of pharmaceutical compounds in seawater has been investigated across various regions of the world, including the Mediterranean Sea, the North Sea, the Adriatic Sea, and the Pacific and Indian Oceans [16,17,18,19,20,21,22,23].

Several classes of pharmaceuticals have been investigated in marine environments, including non-steroidal anti-inflammatory drugs (NSAIDs), analgesics, antibiotics, beta-blockers, lipid regulators, and psychoactive substances [17,22,24,25,26]. Among the most frequently detected NSAIDs and analgesics in seawater from the North Sea, Mediterranean Sea, Adriatic Sea, and even the Pacific and Indian Oceans are diclofenac, ibuprofen, naproxen, ketoprofen, salicylic acid, acetaminophen, and codeine, with concentrations reaching several hundred nanograms per liter (ng/L) [17,18,20,21,24].

Analyzing pharmaceutical compounds in seawater is essential for evaluating water quality, understanding the behavior of chemicals, and assessing the toxicity of these substances on sensitive marine organisms (plants and animals) that form part of the food chain. The main objective of this study is to validate a solid-phase extraction method followed by liquid chromatography coupled with mass spectrometry (SPE-LC-MS/MS) for the detection of non-steroidal anti-inflammatory drugs (NSAIDs) in seawater from the coastal area of the Black Sea. Additionally, the validated method will be applied to investigate the occurrence of analgesic compounds in this region. The method validation will provide a first-time assessment of the pollution levels in Black Sea coastal waters with emerging contaminants and help evaluate the ecological risks posed to aquatic organisms. The chemical properties of the pharmaceutical compounds studied are summarized in Table 1 [27,28].

## 2. Materials and Methods

### 2.1. Chemicals and Materials

The pharmaceuticals that were studied, namely piroxicam (PIR), ketoprofen (KET), naproxen (NAP), indomethacin (IND), diclofenac (DIC), ibuprofen (IBU), acetaminophen (ACE), and caffeine (nerve stimulator, CAF), were obtained from Sigma-Aldrich (Steinheim, Germany). LiChrosolv acetonitrile, methanol, and water for liquid chromatography were purchased from Merck (Darmstadt, Germany). Formic acid (99.9% purity) was supplied by Agilent (Supelco Inc., Bellefonte, PA, USA). SPE Strata-X cartridges (500 mg, 6 mL) used for solid-phase extraction were acquired from Phenomenex (Milford, MA, USA). Basic standard solutions with concentrations of 500 ng/mL were prepared in methanol and stored at −20 °C. Individual dilutions and mixed standard solutions of analytes were prepared in acetonitrile and the initial mobile phase, which consisted of 90% acetonitrile and 0.1% formic acid. All stock solutions and their dilutions stored in the dark at 4 °C.

### 2.2. Water Sampling Sites

**Figure 1:**
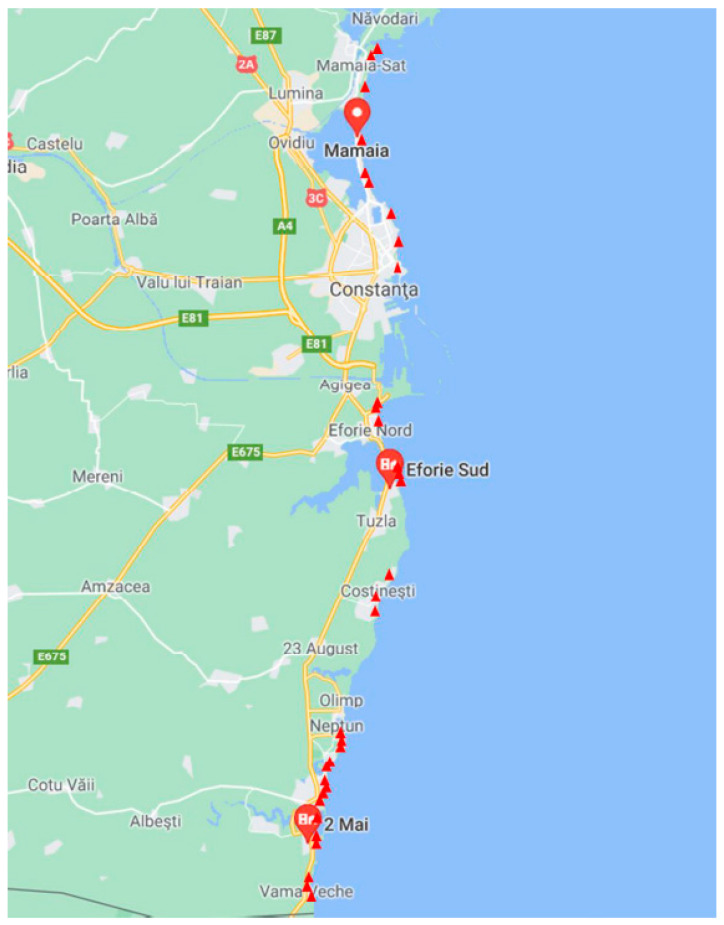
Seawater sampling locations from the coastal area of the Black Sea (sampling depth 30 cm from surface).

The seawater samples were collected from 45 locations along the Black Sea coast in August 2021, during the tourist season, approximately 1 m from the shore. The coordinates of the sampling points are presented in Table 2 and in Figure 1. The samples were taken from the surface (30 cm deep) in 1 L glass bottles and stored in the refrigerator. The samples were taken from 13 beaches in 6 localities: Vama Veche and 2 Mai (villages in Limanu commune); Mangalia (city), Saturn, Venus, Jupiter, Neptun (4 resorts in Mangalia), Eforie South, Eforie North (Eforie city), Costinesti (commune), Navodari (town), Constanta (municipality), Mamaia (resort in the town of Constanta). This is the first nationwide study on the presence of pharmaceutical compounds in the Romanian Black Sea coastal area that led to preliminary results regarding the contamination of seawater with pharmaceutical substances.

The chemical quality of the seawater is influenced both by the quality of the wastewater discharged by treatment plants directly into the sea and by the composition of the water from the Danube that flows into the sea. The Constanța South treatment plant processes approximately 60% of the domestic and industrial wastewater collected by Constanța’s combined sewage system. The treatment process is a mechanical-biological one, operating on two separate lines: fine and coarse screening, fat removal, primary sedimentation, and conventional activated sludge process. The station is designed for 461,000 inhabitants, where the maximum daily flow is 276,480 m^3^/day. The South Eforie Treatment Plant (STP) is located in the city of Eforie South. It receives wastewater from Agigea, Tuzla, Techirghiol, Schitu, Costinesti, and the cities of Eforie North and Eforie South through 14 pumping stations. The sewage system serving the wastewater treatment plant is a unitary system. Station Eforie South is designed for a load of 140,000 EI (inhabitant equivalent) in season (summer) and 69,000 EI in off-season (winter). The installation is sized at the capacities of 64,368 m^3^/day (season) and 27,820 m^3^/day (off-season). The Constanta North Wastewater Treatment Plant has a capacity of 165,888 m^3^/day, designed for 255,000 PE (equivalent population) and ensures wastewater treatment for the northern area of the city and from the Mamaia resort.

### 2.3. Extraction of Pharmaceuticals from Seawater

The samples with visible particles were pre-filtered to remove suspended matter, which could otherwise clog the solid-phase extraction (SPE) cartridge. Filtration was performed with a vacuum filter (Whatman GF/A) using Millipore 0.45 μm glass fiber filter paper (Burlington, MA, USA). The container with the water sample was rinsed twice with 5 mL of double-distilled water. For the NSAIDs extraction of the water samples, a solid-phase extraction (SPE) procedure was employed using a semi-automatic SPE system (Auto-Trace 280, Thermo Scientific, Waltham, MA, USA). Polymeric Strata-X cartridges (500 mg/6 mL, styrene-divinylbenzene polymer) were used for the extraction. The cartridges were conditioned with 10 mL of methanol followed by 10 mL of ultrapure water adjusted to pH 2.0 using hydrochloric acid (HCl, 1:3). Subsequently, 500 mL of the aqueous sample was passed through the SPE cartridges, during which analytes were retained on the sorbent, and the aqueous matrix was discarded. To remove matrix interferences, the cartridges were rinsed with 10 mL of ultrapure water (pH 2.0), then dried with air for 20 min. The retained analytes were eluted with 6 mL of methanol and collected in a concentration tube. The extracts were evaporated to near dryness under a gentle stream of nitrogen using a Biotage II evaporation system in a water bath at 45 ± 5.0 °C. The dried residue was reconstituted with 1 mL of the initial mobile-phase mixture (0.10% formic acid in water/acetonitrile, 90:10, *v*/*v*). If the extract contained visible particles or appeared cloudy, it was filtered through a 0.45 μm PTFE Millipore filter. Finally, 1 mL of the purified extract was introduced into an LC vial for chromatographic analysis.

### 2.4. LC–MS/MS Sample Analysis

The analytical method was based on liquid-phase chromatography (UHPLC 1260 Agilent Technologies, Santa Clara, CA, USA) using the Zorbax Eclipse XDB C18 chromatographic column (100 × 2.1 mm, 3.5 μm dp, Agilent Technologies, Santa Clara, CA, USA) and gradient elution with acetonitrile and formic acid (0.10%). The simultaneous elution of eight pharmaceutical compounds was carried out using a gradient mobile phase starting with 90.0% aqueous formic acid solution (0.10%) and 10.0% acetonitrile (ACN). Over the first 2 min, the acetonitrile content was increased to 50.0% at a flow rate of 0.30 mL/min to facilitate the separation of the most polar analytes—acetaminophen and caffeine. To separate the less polar compounds—piroxicam, ketoprofen, naproxen, indomethacin, diclofenac, and ibuprofen—the acetonitrile concentration was further increased from 50% to 100% between minutes 2.0 and 9.0. During this gradient phase, the compounds were fully separated. A 10 µL volume of each sample was injected, and the chromatographic column was maintained at a constant temperature of 20 °C.

Detection was performed using a 6410 triple quadrupole mass spectrometer (Agilent Technologies) operating in positive electrospray ionization (ESI) mode. Ionization of analyte molecules was conducted at a source temperature of 300 °C, with a nitrogen drying gas flow rate of 8.0 L/min, a nebulizer pressure of 40 psi, and a capillary voltage of 4000 V. For each analyte, a specific ion transition was monitored, corresponding to the precursor ion and its most abundant product ion. The most intense transition was used for both quantification and confirmation. Data acquisition was performed in multiple reaction monitoring (MRM) mode to enhance sensitivity and selectivity. To assess possible interference from the sample preparation and analysis process, a blank sample was included in each analytical batch. None of the target compounds were detected in the control samples. All analyses were performed in triplicate.

## 3. Results and Discussion

### 3.1. SPE-LC-MS/MS Method Development

A liquid chromatography method (LC-MS/MS) was developed using the separation of eight pharmaceutical compounds (seven NSAIDs on one nervous stimulant, caffeine) on Zorbax Eclipse SDB C18 (100 × 2.1 mm, 3.5 μm) at a flow rate of 0.30 mL/min. The good separation of the compounds (with good chromatographic resolutions) was achieved with an optimal gradient of the mobile phase that allowed the elution of the analytes at different retention times (Table 3).

From minute 9.01 to minute 14.01, the mobile phase was operated in a gradient from 100% ACN to 10% ACN with a flow rate of 0.50 mL/min to re-equilibrate the chromatographic column and prepare the chromatograph for the next injection.

First, the compounds were injected individually from standard solutions of 5.0 mg/L to obtain molecular precursor ions, recording the spectrum of the parent molecular ion M-H^+^. To achieve optimal sensitivity, dedicated collision energies and fragmentation settings were applied to generate the most abundant molecular transitions from daughter ions to parent ions. These parameters were carefully optimized to produce the highest signal intensities for each compound, thereby minimizing the quantification limits.

**Figure 2:**
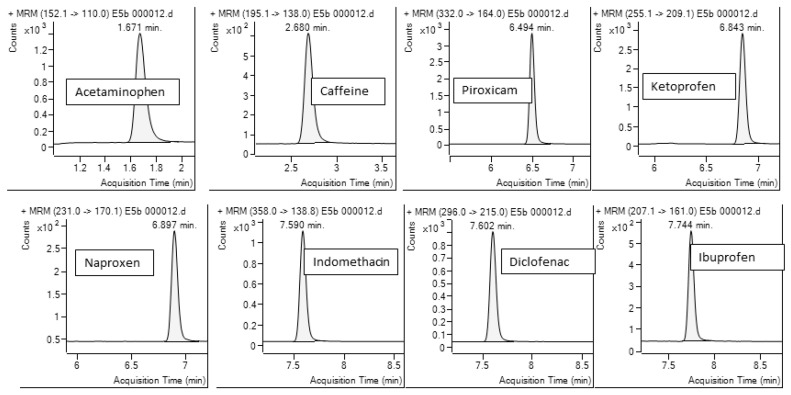
ESI MRM chromatogram of a standard calibration containing all analytes 50.0 ng/L.

The optimal fragmentation energies, MRM transitions, and collision energies (CE) for each analyte are detailed in Table 4. To further enhance the sensitivity and selectivity of the method, six time segments were established within the MRM acquisition window. Each analyte was assigned to its corresponding segment based on its chromatographic retention time, allowing for more efficient signal acquisition and reduced background noise. For acetaminophen, the ion transition from mass 152.1 to 110 was acquired, for caffeine the MRM transition was monitored from the ion with mass 195.1 to the ion 138. Fragmentation voltages and dedicated collision energies were applied to each segment to obtain transitions from the precursor ions to the ions produced according to Table 4, Figure 2.

**Figure 3:**
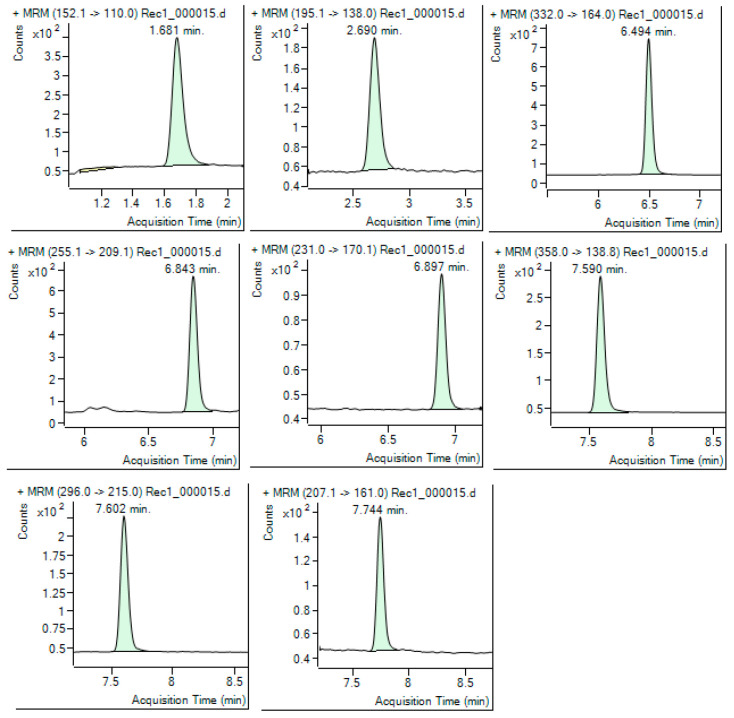
ESI MRM Chromatogram of a seawater sample fortified at 100 ng/L for the calculation of recovery rate.

Figure 3 shows the chromatogram for a water sea sample fortified with known standard concentrations (100 ng/L) used to determine the recovery of analytes.

**Figure 4:**
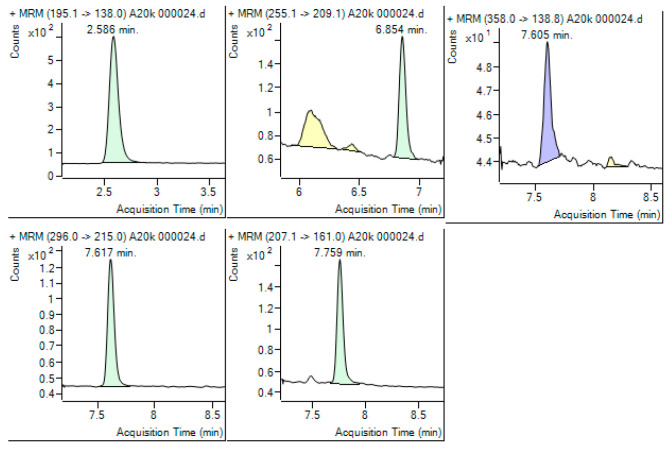
Chromatogram of a seawater sample extracted showing the presence of caffeine, ketoprofen, indomethacin, diclofenac, and ibuprofen.

In Figure 4, the detection of some pharmaceutical compounds (caffeine, ketoprofen, indomethacin, diclofenac, and ibuprofen) in a seawater sample is depicted.

### 3.2. Performance Parameters of the Method

Calibration was performed using an external standard method with six concentration levels ranging from 1.00 to 100 ng/mL. Chromatograms were recorded for each level, and calibration curves were constructed using MassHunter B.07 software. The chromatographic peak area was plotted on the *y*-axis (ordinate) against the analyte concentration on the *x*-axis (abscissa). A six-point calibration curve was obtained for each analyte within this concentration range. The lower limit of linearity was approximately 1.00 ng/mL for all compounds. The main performance parameters, including calibration data, are presented in Table 5. Linearity was evaluated using the correlation coefficient (R^2^), with values ranging from 0.994 to 0.999, indicating excellent linearity across the tested concentration range. Two key validation parameters—limit of quantification (LOQ) and analytical recovery—were assessed to confirm the presence of analytes in water samples. The LOQ was defined as the concentration at which the signal-to-noise (S/N) ratio was equal to 10. This was determined using chromatograms from fortified water samples spiked with a standard solution (1.00 ng/L) and subjected to the same extraction procedure. The LOQs for the analyzed compounds in the water matrix ranged from 0.100 ng/L for piroxicam to 1.50 ng/L for ibuprofen, demonstrating high sensitivity of the method.

To ensure accurate quantification, the analytical precision of the method was evaluated at a concentration level of 50.0 µg/L under both short-term and long-term conditions. Repeatability was assessed by analyzing three sub-samples of seawater spiked with the standard on the same day, while intermediate precision was evaluated by repeating the analysis over three consecutive days under the same experimental conditions. Precision was expressed as the relative standard deviation (RSD%). The RSD values ranged from 4.90% for diclofenac to 16.5%, as presented in Table 5. All values were within the generally accepted limit of ≤20.0% RSD for LC-MS methods, indicating satisfactory method precision.

To assess the recovery efficiency of the target analytes, a seawater sample was analyzed both with and without the addition of a known standard concentration (50.0 ng/mL). Specifically, 1.00 mL of the 50.0 ng/mL calibration solution was added to 0.500 L of seawater. A separate unfortified sample was also analyzed under the same conditions, and the background concentrations detected were subtracted from the spiked sample results to determine true recovery. Both samples were processed using the developed solid-phase extraction and LC-MS method. According to the validation criteria, the acceptable recovery values should fall within ±30.0% of the true concentration. The method demonstrated satisfactory recovery yields for all investigated compounds, ranging from 76.5% for piroxicam to 95.1% for indomethacin, confirming the reliability of the extraction and quantification procedure. The matrix effect was determined using the method of fortification of the sample with a post-extraction working standard. The signal obtained from the post-extraction fortified sample was compared with the signal of a working standard used for fortification.

The environmental fate of pharmaceutical compounds can be inferred from their physicochemical properties, particularly their hydrophobicity. The octanol–water partition coefficient (log Kow) is commonly used to estimate a compound’s affinity for organic matter (e.g., sediments) versus its solubility in water. Compounds with log Kow < 1.0 are considered hydrophilic and tend to remain dissolved in the aqueous phase, exhibiting high mobility in aquatic environments. For example, caffeine, with a log Kow of –0.55, is highly water-soluble. In contrast, compounds with log Kow > 4.0 are typically hydrophobic, showing low environmental mobility and a high tendency to adsorb to sediments or particulate matter. Pharmaceuticals with intermediate hydrophobicity (log Kow between 1.0 and 4.0) demonstrate moderate mobility and can partition between both the aqueous and solid phases. While log Kow is a useful descriptor for neutral molecules, many non-steroidal anti-inflammatory drugs (NSAIDs) are ionized at environmental pH levels (e.g., pH 7.2), often carrying a negative charge. In such cases, the octanol–water distribution coefficient (log Dow)—which accounts for ionization—is a more appropriate predictor of environmental behavior, as it varies with pH.

**Figure 5:**
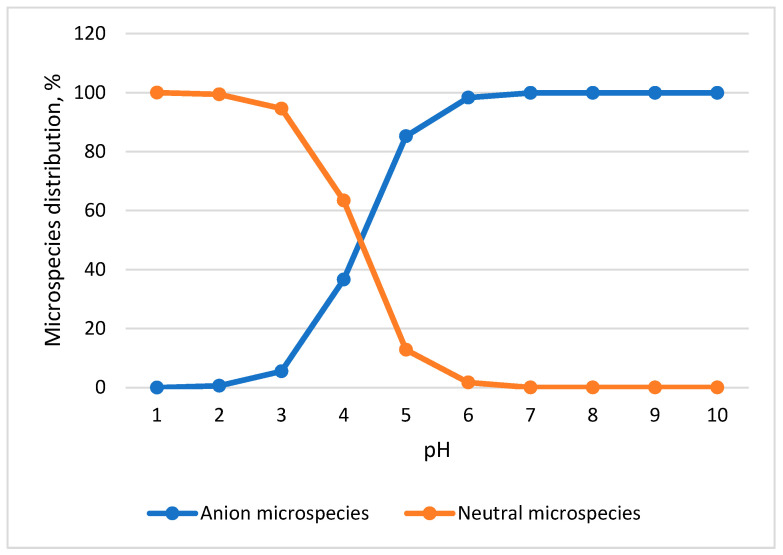
Naproxen microspecies distribution dependent of pH.

At pH 2, the selected pharmaceutical compounds (log Dow −0.38 ÷ 4.25) are mostly present in non-ionic form and show maximum interaction with the polymeric adsorbing phase Strata X being retained inside it, generating maximum recoveries. In contrast to the neutral pH of sea samples (7.20), the analytes (log Dow −1.10 ÷ 1.52) are present in negatively ionized form and will not be retained quantitatively in the adsorbent used for solid-phase extraction. Therefore, the pH of the seawater samples was adjusted to 2.00 in order to enhance analyte recovery and ensure optimal extraction efficiency. In order to optimize the extraction in the solid phase of the analytes from seawater samples, the pH of the samples and the type of adsorbent (polymeric styrene divinyl benzene—Strata X and octadecyl silica—Strata C18) were studied. The recovery of the analytes was studied at neutral pH (of environmental samples) and at acidic pH (2.00) due to the assumption that the anti-inflammatory compounds are non-ionized (neutral) at acidic pH and will interact maximally with the adsorbent (Figure 5). At pH 2.00, the recovery was higher (76.5–95.1%) compared to the recovery at pH 7.20 (37.2–60.7%, Table 6). The recoveries obtained at acidic pH verify the proposed hypothesis that analytes are neutral at this pH value and ensure maximum recoveries. On the other hand, in the case of using two types of adsorbents, it was found that the recoveries calculated with Strata C18 are lower (27.7–61.4%) than those obtained with polymeric adsorbent (76.5–95.1%), proving the efficiency of the polymeric material in the extraction of anti- inflammatory agents from the seawater samples.

### 3.3. Occurrence of Pharmaceuticals in Seawater Samples

**Figure 6:**
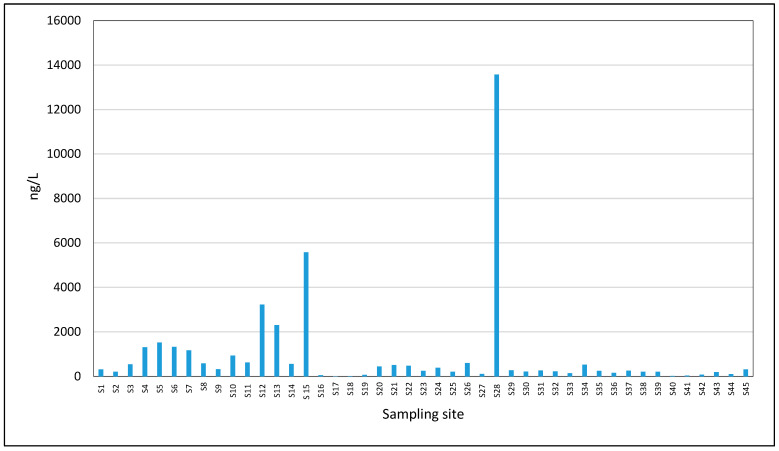
The spatial profile of caffeine in all the locations studied.

Of the eight pharmaceutical compounds analyzed, acetaminophen, piroxicam, and indomethacin were not detected in any of the seawater samples probably due to low usage but also the dilution factor due to seawater. In contrast, caffeine was detected in 100% of the samples, making it the most prevalent and dominant compound (see Table 7). The next most frequently detected compound was ibuprofen, present in 42.9% of samples, followed by ketoprofen (30.9%), diclofenac (23.8%), and naproxen (21.4%). The maximum concentrations observed ranged from 13.4 ng/L for ketoprofen to 13,575 ng/L for caffeine (Figure 6), indicating significant variability in occurrence and concentration among the detected compounds. The order of decreasing maximum concentrations of pharmaceutical compounds in the water of the Black Sea, the coastal area, was CAF > IBU > NAP > DIC > KET.

In recent years, numerous studies have investigated the presence of analgesic compounds in seawater across various regions of the world. Table 8 summarizes the findings of similar research conducted in different countries, providing a basis for comparison with the results obtained in this study.

It is important to emphasize that sampling and analysis of the seawater were conducted in August, a peak tourist season along the Black Sea coast. During this period, the increased consumption of caffeinated beverages such as coffee—commonly served in local food establishments—contributes to elevated caffeine levels in wastewater, which is discharged into the marine environment through local treatment plants. Although caffeine is a naturally occurring substance found in coffee, tea, cocoa, and kola nuts, and is frequently added to beverages, its presence in environmental waters is primarily attributed to domestic sewage discharges. This results from incomplete removal by conventional wastewater treatment systems. Due to its high water solubility (13.5 g/L), caffeine is expected to persist in aquatic environments. Elevated concentrations have been correlated with densely populated areas and tourism-related activities. Reported caffeine concentrations in various water sources include surface waters (112–781 ng/L), wastewater/effluents (0.07–126 µg/L), and coastal waters (15.0–185 ng/L) [33,34,35]. In the present study, caffeine concentrations in Black Sea coastal seawater were found to be equal to or higher than those reported in other marine environments, such as the Mediterranean Sea (327 ng/L) and the Red Sea (7708 ng/L), underscoring the impact of seasonal population influx on local water quality [30,32].

**Figure 7:**
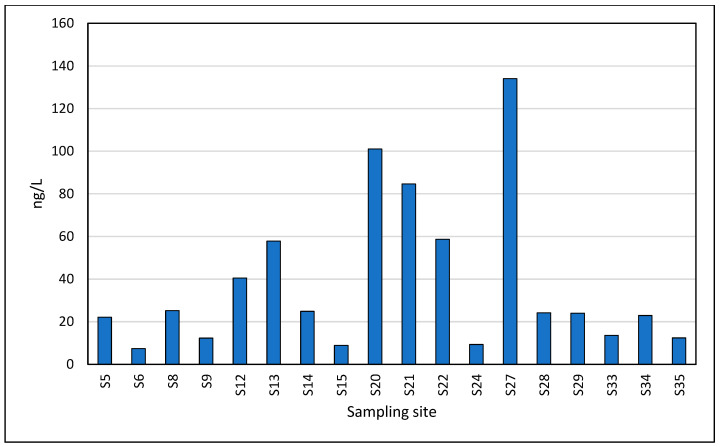
Spatial profile of Ibuprofen in the studied locations.

The next compound detected with decreasing concentration values was ibuprofen, which showed a maximum of 134 ng/L in the S27 Eforie North area, followed by 101 ng/L in S20 Neptun (Figure 7). This compound is intensively used in the treatment of inflammatory conditions as an analgesic, anti-inflammatory and antipyretic. Ibuprofen was detected in a concentration range of 7.40–134 ng/L, with an average concentration value of 37.9 ng/L.

The elevated concentrations of pharmaceutical compounds detected in the study area can be attributed to anthropogenic pressures, particularly in coastal zones with significant human activity. In the city of Eforie, for instance, treated effluents from the local sewage treatment plant are discharged directly into the coastal waters, contributing to the contamination observed at these sampling sites. Caffeine, known for its high solubility and persistence, can be conservatively transported through river systems, eventually reaching the Black Sea via the Danube River. Its widespread occurrence in this study highlights both local wastewater inputs and regional transport pathways. Ibuprofen (IBU), the third most consumed pharmaceutical globally, also poses considerable environmental concern. It enters aquatic systems primarily through human excretion following pharmaceutical use. Approximately 15.0% of ibuprofen is excreted unchanged, either as the parent compound or conjugated forms (e.g., glucuronide and thiol derivatives), along with several active metabolites such as carboxy-ibuprofen and hydroxy-ibuprofen. Conventional wastewater treatment technologies are generally ineffective at fully removing ibuprofen, with reported removal efficiencies ranging from 12.0% to 100%, depending on the treatment system design and operational conditions [35]. This incomplete removal leads to its persistent presence in various water bodies. In the present study, IBU concentrations were found to be comparable to levels reported in the Atlantic Ocean (222 ng/L), lower than those in the Adriatic Sea (1143 ng/L), and lower than the values measured in the Mediterranean Sea (1500 ng/L) [17,31]. These findings further confirm ibuprofen’s ubiquitous occurrence in marine environments and the need for improved wastewater treatment solutions and pharmaceutical usage management, especially in densely populated or tourist-heavy coastal areas.

**Figure 8:**
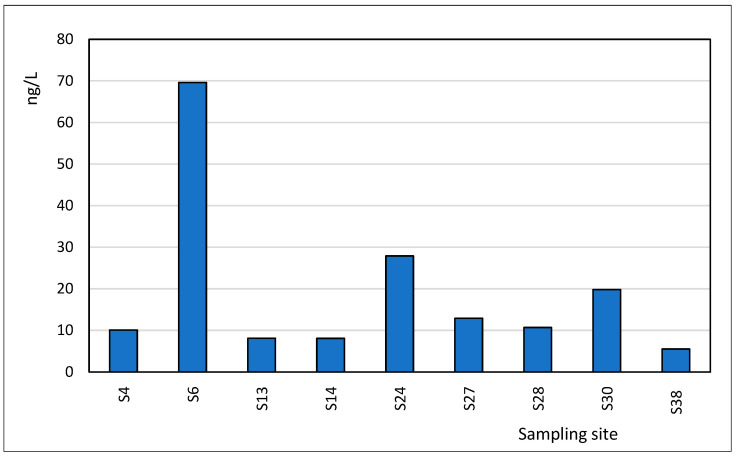
Spatial profile of Naproxen in the studied locations.

Another analgesic detected was naproxen, which showed a maximum concentration of 69.6 ng/L in point S6, area 2 Mai, followed by 27 ng/L in S24, Eforie South. This compound presented a range between 5.50 ng/L and 69.6 ng/L (Figure 8). The average concentration observed in the case of this compound was 19.2 ng/L. Naproxen is widely used globally as a non-steroidal anti-inflammatory drug (NSAID) to treat pain, inflammation, and fever. After human consumption, the parent compound and its metabolites are excreted and enter wastewater distribution systems, eventually reaching wastewater treatment plants (WWTPs). Due to variable removal efficiency in conventional treatment systems, naproxen can persist and be discharged into receiving water bodies, contributing to pharmaceutical pollution in coastal and marine environments.

The removal efficiency of naproxen in wastewater treatment plants (WWTPs) varies significantly, with reported rates ranging from 55.0% to 98.0%. Consequently, low concentrations of naproxen (NAP) and its metabolite 6-O-desmethylnaproxen (D-NAP), typically in the nanogram to microgram per liter range, are often detected in receiving waters, including rivers and, ultimately, the Black Sea. In effluent from WWTPs, naproxen concentrations have been reported to range from 25.0 ng/L to 33.9 μg/L [36]. These concentrations are influenced by several factors, including the physicochemical properties of naproxen, such as solubility and chemical stability, as well as environmental conditions. The mobility of naproxen in the environment is primarily governed by its chemical properties, such as its dissociation constant and octanol-water partition coefficient (log Kow). The log Kow value of 3.20 indicates that naproxen is hydrophobic and has a tendency to be adsorbed by suspended particles, sediment, or sewage sludge. Thus, its fate in the environment is heavily influenced by two key phenomena: sorption and degradation. The sorption process, which involves the attachment of naproxen to particles, is inversely related to pH. Since naproxen contains a carboxylic acid group, which becomes deprotonated in the environmentally relevant pH range of 5.0–8.0, it predominantly exists in an anionic form in natural waters. This anionic form enhances naproxen’s potential to conjugate with basic substances in aquatic environments.

Comparable concentrations of naproxen have been reported in other marine environments, such as the Mediterranean Sea (Spain), where levels of 95.8 ng/L were detected, further highlighting the widespread presence of naproxen in coastal waters [17]. France reported much higher values of naproxen in the Mediterranean Sea of 2000 ng/L [17,19].

**Figure 9:**
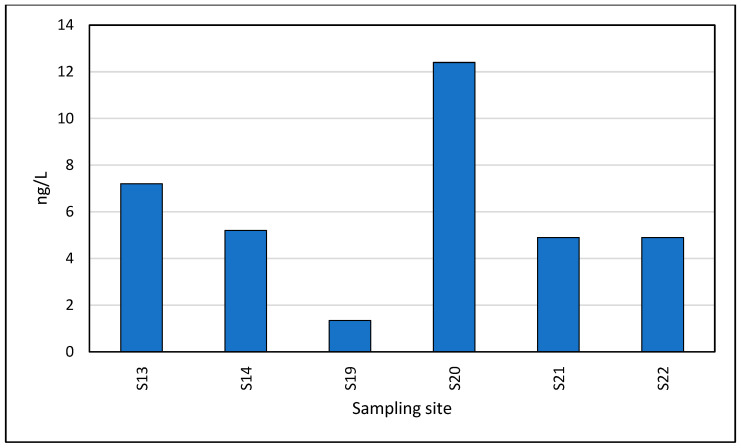
Spatial profile of Ketoprofen in the studied locations.

Ketoprofen was detected in lower concentrations than the other analgesics: 1.30–13.7 ng/L, the maximum being observed in the Neptun S20 area, followed by 7.20 ng/L in samples from the Saturn S13 area (Figure 9).

The concentrations of ketoprofen in seawater samples from various regions show significant variability. For example, values in the Indian Ocean have been reported to range from <1.70–6.60 ng/L, while the Mediterranean Sea has seen concentrations around 2.60 ng/L [32]. In comparison, other studies have documented much higher levels of ketoprofen, such as 89.7 ng/L in the Atlantic Ocean [19] and 6000 ng/L in the Mediterranean Sea [31]. As a non-steroidal anti-inflammatory drug (NSAID), ketoprofen has a hydrophilic character due to its log Kow, which indicates a lower tendency to adsorb to organic matter, making its removal through sorption processes in sewage treatment plants ineffective. Instead, its elimination largely depends on the type of chemical or biological treatment used. This inefficiency in conventional treatment systems leads to highly variable removal rates, ranging from 15% to 98%. As a result, ketoprofen remains in water bodies at concentrations ranging from nanogram to microgram per liter levels. Given its persistence in the environment, ketoprofen is detected at significant concentrations in receiving waters, contributing to pharmaceutical contamination in coastal and marine ecosystems.

**Figure 10:**
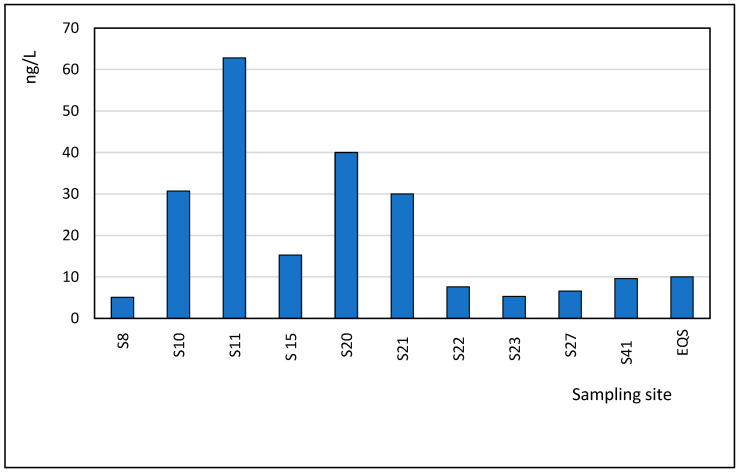
Spatial profile of Diclofenac in the studied locations.

Diclofenac (DIC), a widely used analgesic and anti-inflammatory drug, was detected in seawater samples from the Black Sea at concentrations ranging from 5.10 ng/L to 62.8 ng/L. The highest concentrations were recorded in the Mangalia area (62.8 ng/L) and Neptun (40.0 ng/L), as shown in Figure 10. Diclofenac is commonly found in wastewater, effluents, and surface waters, as it is extensively used to treat inflammatory conditions. Most diclofenac is metabolized in the human body, and less than 1.0% of the administered dose is excreted as un-metabolized DIC. In recognition of its environmental impact, diclofenac was added to the EU Watch List of substances requiring environmental monitoring in 2015 [37]. During the review, an Environmental Quality Standard (EQS) of 100 ng/L for inland waters and 10 ng/L for coastal waters was proposed for diclofenac. Notably, in this study, 5 out of 45 samples (11.1%) from the Black Sea exceeded the proposed coastal water standard of 10 ng/L. Diclofenac is known to have toxic effects on various species in the environment, even at concentrations as low as ≤ 1.0 μg/L. In terms of its removal in sewage treatment plants (STPs), the elimination rate is relatively low, varying between 20 and 50% [38]. Comparisons with other studies show that diclofenac concentrations in similar marine environments are consistent with the findings in this study. For example, concentrations in the Mediterranean Sea ranged from 5.50 ng/L to 69.6 ng/L, while in the Atlantic Ocean, concentrations ranged from <0.02 ng/L to 241 ng/L. Notably, higher values have been reported, such as 1500 ng/L in the Mediterranean Sea, which is much higher than those observed in the current study [19,31,32].

In aquatic environments, pharmaceuticals are subject to sorption processes onto sediments, which are influenced by both the properties of the sediment—such as organic matter content and pH—and the physicochemical characteristics of the pharmaceutical compounds, including the dissociation constant (pKa) and the octanol-water partition coefficient (log Kow). According to the classification by Rogers [39], compounds with a log Kow below 2.5 exhibit low sorption potential, values between 2 and 4 indicate medium sorption, while log Kow above 4 suggests high sorption potential. In this context, the log Kow values of diclofenac (DIC) and ibuprofen (IBU) are 4.51 and 3.97, respectively. Based on these values, diclofenac can be expected to exhibit high sorption, whereas ibuprofen demonstrates medium sorption, which explains their detection in both water and sediment samples. However, sorption behavior is also affected by the ionization state of the compounds, which is pH-dependent. The pKa values of diclofenac (3.99–4.3) and ibuprofen (4.45–5.3) suggest that, at the typical seawater pH (~7.2), both compounds would predominantly exist in their negatively ionized (anionic) forms. This reduces their sorption affinity to surface sediments despite their log Kow values, leading to greater mobility in the water column and reduced binding to particulate matter [40].

### 3.4. Ecological Risk Assessment

Several studies have demonstrated that non-steroidal anti-inflammatory drugs (NSAIDs) can act as endocrine disruptors in aquatic organisms, particularly fish. Gravel and Vijayan were the first to report that exposure to ibuprofen (IBU) disrupted cortisol production in rainbow trout (*Oncorhynchus mykiss*), indicating interference with the stress response system of the fish [41]. In another study, Corcoran et al. found that ibuprofen significantly induced CYP2K expression in hepatocyte cultures derived from common carp (Cyprinus carpio), suggesting that NSAIDs may alter hepatic metabolic pathways and endocrine function in fish [42].

The environmental risks posed by the pharmaceutical compounds were evaluated by calculating risk quotients (RQs), using toxicological data reported in the literature. The assessment was conducted according to standard risk evaluation practices, using the following equations:

PNEC = NOEC/AF,(1)

RQ = MEC/PNEC,(2)

where PNEC—no effect concentration; NOEC—no observed effect concentration; AF—rating factor of 100 and 1000 in chronic and acute toxicity, respectively; and MEC—the maximum concentration of pharmaceutical obtained in this study. The risk quotient (RQ) method was applied as a novel approach to estimate the environmental risk posed by the pharmaceuticals most frequently detected in seawater samples. This method enables a preliminary screening of potential ecological threats and supports the prioritization of contaminants for further monitoring and management efforts.

The RQ values were calculated to assess the ecological risk of pharmaceutical contaminants to aquatic organisms across various trophic levels, including algae, crustaceans, invertebrates, and fish. The calculations were based on measured environmental concentrations (MECs) obtained in this study, and predicted no-effect concentrations (PNECs) derived from published toxicological endpoints (e.g., EC_50_, NOEC), adjusted using appropriate assessment factors (AFs). Detailed information regarding the test species, toxicological endpoints, assessment factors, MEC, and the resulting PNEC values used for RQ determination is provided in Table 9. The RQ values were interpreted using established risk classification criteria [43]: RQ < 0.01, unlikely to pose risk; 0.01 ≤ RQ < 0.10 low risk; 0.10 ≤ RQ < 1.0 medium risk; RQ ≥ 1.0 high risk.

By evaluating the ecological risk that the pharmaceutical compounds pose on the biota in the aquatic environment, it was observed that IBU presents a high risk for the echinoderm *Parocentrotus lividus* (RQ 1340) and for the crustacean *Carcinus maena* (RQ 2.68). In addition, the CAF that showed a very high value of 13,575 µg/L generates a high ecological risk for the crustacean *Daphnia magna* (RQ 2610) and for the fish *Pimephales promelas* (RQ 2610). At the same time, CAF presented a medium risk for the *Ceriodaphnia dubia* flea (RQ 1.35). By comparing with the values reported in the literature, it was observed that the results differ in the case of some drugs.

Previous studies have highlighted the environmental risks posed by NSAIDs in various aquatic ecosystems. For example, ibuprofen (IBU) was found to pose a medium risk (0.10 ≤ RQ < 1.0) to aquatic organisms in the Yellow River and Liao River, while diclofenac (DIC) posed a medium risk in the Hai River and a high risk (RQ ≥ 1.0) in both the Yellow and Liao Rivers [55]. Similarly, high RQ values for DIC, IBU (consistent with the findings of the present study), and naproxen (NAP) have been reported in Danish and Norwegian aquatic environments [56,57]. Due to its widespread occurrence and potential ecotoxicological effects, diclofenac has been added to the watch list under the EU Water Framework Directive, aimed at gathering more comprehensive data on its presence and impact in the environment [58].

Adenaya et al. reported the presence of ibuprofen in the North Sea at concentrations ranging from 9.20 to 52.1 ng/L, with a detection frequency of 100%, indicating its widespread occurrence in coastal waters. Additionally, German researchers have observed that ibuprofen poses a high ecological risk to the marine diatom *Skeletonema marinoi*, particularly in the Jade Bay and the Weser estuary, highlighting its potential to adversely affect primary producers in marine ecosystems [59].

Results from whole-organism toxicity studies have indicated that the environmental concentrations of several NSAIDs—such as diclofenac (DIC), naproxen (NAP), and ketoprofen (KET)—are generally well below levels known to cause acute toxicity in marine organisms [28]. Based on these findings, these compounds do not currently pose a significant direct toxicological threat to seawater organisms at the concentrations observed in the environment. However, certain pharmaceuticals, notably ibuprofen (IBU) and caffeine (CAF), require special attention due to their reported endocrine-disrupting potential, toxicity, and in some cases, bioaccumulative behavior. These properties may lead to chronic ecological impacts, even at low concentrations. Therefore, IBU and CAF should be prioritized in future monitoring and risk management strategies, particularly in marine ecosystems along the Black Sea coast, where both compounds were frequently detected.

Recently, researchers from Italy detected anti-inflammatory compounds such as ketoprofen (0–57.5 ng/g) and diclofenac (0–6.7 ng/g) in Parazoanthus axinellae from the North Sea, indicating the potential for these compounds to bioaccumulate in anthozoans [60].

On the other hand, ibuprofen (IBU) has also demonstrated bioaccumulative behavior in marine organisms. In the Sea of Marmara, ibuprofen was the most frequently detected pharmaceutical in biota tissue samples, with concentrations ranging from <3.0 to 1225 ng/g dry weight (dw) [61].

### 3.5. Correlation Between Target Pharmaceuticals in the Romanian Seawater

The Spearman correlation was used to understand the relationships between the analytes and their sources of origin in seawater samples. The Spearman correlations showed a specific pattern between the contaminations investigated in the water matrix. Thus, significant correlations were obtained between certain groups of substances. Positive correlations were observed for NAP: DIC (r 0.975) and DIC: KET (r 0.929), highlighting that these compounds come from similar sources or transfer processes. The negative correlation coefficients may suggest that the analytes do not come from the same source of contamination (the same sewage effluent discharged into the Black Sea) but from different sewage treatment plants (Constanta, Navodari, Mangalia, Eforie North). Also, the negative correlations obtained for the other compounds may be due to the dilution effect of the Black Sea in their case, or the effects of their degradation into by-products, or some differentiated elimination efficiencies in the treatment plants.

## 4. Conclusions

For the first time in Romania, eight pharmaceutical compounds from several chemical classes (analgesic, anti-inflammatory, antipyretic, and nerve stimulant) were studied in the water of the Black Sea along the coast of Constanta County. High concentrations of pharmaceuticals were detected in seawater samples, with levels ranging from 1.3 ng/L for ketoprofen to 13,575 ng/L for caffeine (in the Costinești area). Caffeine emerged as the dominant and ubiquitous compound in the seawater samples, being detected in all samples. Ibuprofen was the most frequently detected compound, present in 42.9% of samples, followed by ketoprofen (31.0%), diclofenac (23.8%), and naproxen (21.4%). The large values of concentrations determined in the case of these contaminations can be due to the effluent discharged by the Constanta South, Constanta North, Eforie, Mangalia cities wastewater treatment stations into the Black Sea. By evaluating the spearman correlation indices, it was observed that three compounds (NAP: DIC, DIC: KET) presented a similar pattern regarding the same contamination source. Through the ecological risk assessment, it was observed that both caffeine and ibuprofen can generate high ecological risks for some echinoderms, crustaceans, and fish. Thus, it is necessary to pay close attention to the monitoring of these pharmaceutical compounds in the aquatic environment due to the aquatic toxicity and the potential synergistic effects generated by complex mixtures of persistent and bio-accumulative organic contaminants.

## Figures and Tables

**Table 1:** Chemical properties of the pharmaceuticals studied.

Compound	Structural Formula	Molecular Mass, g/mol	Log Kow	Log Dow (pH 2)	Log Dow (pH 7.2)	pKa
Acetaminophen	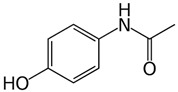	151.06	0.46		0.90	9.38
Piroxicam (PIR)	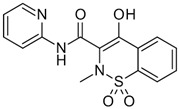	331.06	3.06	−0.38	−1.11	6.3
Ketoprofen (KET)	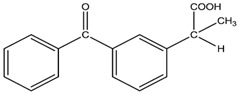	254.09	3.12	3.6	0.58	4.45
Naproxen (NAP)	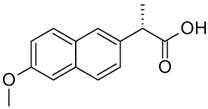	230.09	3.18	2.98	0.12	4.15
Indometacin (IND)	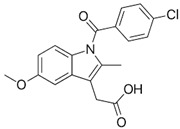	379.05	4.50	3.53	0.78	0.91
Diclofenac (DIC)	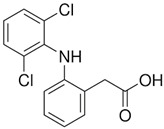	295.01	4.51	4.25	1.23	4.15
Ibuprofen (IBU)	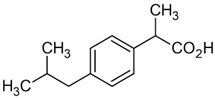	206.13	3.97	3.84	1.52	5.20
Caffeine (CAF)	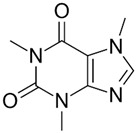	194.19	−0.55	−0.54	−0.54	14.0; 10.4

**Table 2:** Locations of seawater sample collection points (sampling depth 30 cm from surface).

Location	Sample Code	Latitude (N)	Longitude (E)	Location	Sample Code	Latitude (N)	Longitude (E)
Vama Veche	S1	43,748	28,578	Eforie North	S24	44,047	28,649
Vama Veche	S2	43,750	28,577	Eforie North	S25	44,049	28,644
Vama Veche	S3	43,755	28,575	Eforie North	S26	44,048	28,644
2 Mai	S4	43,784	28,580	Eforie North	S27	44,047	28,645
2 Mai	S5	43,786	28,580	Costinesti	S28	44,070	28,641
2 Mai	S6	43,789	28,581	Costinesti	S29	44,068	28,641
Mangalia	S7	43,818	28,589	Costinesti	S30	44,066	28,642
Mangalia	S8	43,811	28,587	Navodari	S31	44,299	28,627
Mangalia	S9	43,813	28,587	Navodari	S32	44,303	28,628
Mangalia	S10	43,814	28,588	Navodari	S33	44,305	28,629
Mangalia	S11	43,815	28,588	Navodari	S34	44,307	28,631
Saturn	S12	43,835	28,591	Navodari	S35	44,309	28,631
Saturn	S13	43,833	28,591	Navodari	S36	44,311	28,632
Saturn	S14	43,831	28,591	Navodari	S37	44,314	28,633
Venus	S15	43,879	28,607	Navodari	S38	44,315	28,634
Jupiter	S16	43,878	28,607	Navodari	S39	44,317	28,635
Jupiter	S17	43,877	28,607	Constanta	S40	44,179	28,658
Neptun	S18	43,888	28,611	Constanta	S41	44,181	28,657
Neptun	S19	43,887	28,611	Constanta	S42	44,192	28,657
Neptun	S20	43,951	28,639	Mamaia	S43	44,236	28,627
Eforie South	S21	43,951	28,639	Mamaia	S44	44,260	28,622
Eforie South	S22	43,950	28,638	Mamaia	S45	44,280	28,622
Eforie South	S23	44,024	28,657				

**Table 3:** Gradient elution program, determined experimentally for the separation of analgesic compounds.

Time (Minute)	Solvent B, Acetonitrile, (%)	HCOOH (A), 0.10%	Flow Rate (mL/min)
0	10.0	90.0	0.30
2.00	50.0	50.0	0.30
6.00	100	0	0.30
9.00	100	0	0.30
9.01	100	0	0.50
14.01	10.0	90.0	0.50
14.50	10.0	90.0	0.30

B: acetonitrile; A: formic acid 0.10%.

**Table 4:** MRM transitions and mass spectrometry operating parameters for the simultaneous analysis of selected analgesics (ESI + positive ionization).

Compound	Retention Time (min)	MRM Transition	Fragmentor Voltage (V)	Collision Energy (V)	Dwell Time (msec)	Cell Accelerator Voltage (V)
Acetaminophen	1.69	152.1→110	90	15.0	200	7.00
Caffeine	2.70	195.1→138	80	20.0	200	7.00
Piroxicam	6.49	332→164	135	15.0	100	5.00
Ketoprofen	6.84	255.1→209.1	140	10.0	100	6.00
Naproxen	6.89	231→170.1	110	30.0	100	7.00
Indomethacin	7.59	358→138.8	135	10.0	100	7.00
Diclofenac	7.61	296→215	75	20.0	100	8.00
Ibuprofen	7.74	207.1→161	110	5.00	100	5.00

**Table 5:** Method recoveries (*n* = 3) and quantification limits (LOQs) of the method for the target organic pharmaceutical contaminants in seawaters.

Compound	R^2^	LOQ (ng/L)	RSDr (%)	RSDR (%)	Recovery (%)	Matrix Effect, %
Acetaminophen	0.996	1.30	7.09	13.7	80.4	77.3
Caffeine	0.994	1.20	8.01	12.2	82.0	87.2
Piroxicam	0.999	0.10	7.48	10.4	76.5	79.4
Ketoprofen	0.999	1.10	8.50	16.5	86.5	88.4
Naproxen	0.999	1.40	6.90	15.6	86.9	90.2
Indometacin	0.997	0.20	5.90	13.6	95.1	80.1
Diclofenac	0.999	0.80	4.90	13.8	84.2	78.7
Ibuprofen	0.999	1.50	5.20	14.6	88.3	82.5

**Table 6:** The recoveries determined in the pH adjustment study and the use of two types of adsorbents, %.

Compound	Strata X, pH 2	Strata X, pH 7.2	Strata C18, PH 2
ACE	80.4	60.7	37.3
CAF	82.0	48.3	27.7
PIR	76.5	37.7	29.5
KET	86.5	55.9	35.2
NAP	86.9	41.7	47.3
IND	95.1	37.2	44.2
DIC	84.2	44.1	61.4
IBU	88.3	51.2	37.3

**Table 7:** Concentration range (ng/L), average concentration values and detection frequencies (%) of the 8 pharmaceutical compounds in seawater.

Compound	Frequency Detection, %	Minimum	Maximum	Average
Caffeine	100	7.90	13, 575	936
Ketoprofen	30.9	1.34	13.7	6.25
Naproxen	21.4	5.50	69.6	19.2
Diclofenac	23.8	5.10	62.8	21.3
Ibuprofen	42.9	7.40	134	38.0

**Table 8:** Concentrations of anti-inflammatory compounds, analgesics present in seawater worldwide.

Compound	Seawater	Country	Concentration, ng/L	Reference
Acetaminophen	Mediterranean Sea	Spain	23.0	[17]
		France	200,000	[19]
	Red Sea	Saudi Arabi	2363	[29,30]
	Atlantic Ocean	Portugal	51.2–584	[31]
	Black Sea	Romania	nd	This study
Diclofenac	Mediterranean Sea	Spain	4.0	[17]
		France	1500	[19]
		Spain	31.9	[32]
	Atlantic Ocean	Portugal	<0.02–241	[31]
	Black Sea	Romania	5.10–62.8	This study
Ibuprofen	Mediterranean Sea	Spain	16.0	[17]
		France	1500	[19]
	Adriatic Sea	Italy	<0.05–1143	[20]
	Atlantic Ocean	Portugal	<0.08–222	[31]
	Black Sea	Romania	7.40–134	This study
Ketoprofen	Mediterranean Sea	France	6000	[19]
		Spain	2.60	[32]
	Indian Ocean	Taiwan	<1.70–6.60	[22]
	Atlantic Ocean	Portugal	<0.30–89.7	[31]
	Black Sea	Romania	1.34–13.7	This study
Naproxen	Mediterranean Sea	Spain	6.0	[17]
			95.8	[32]
		France	2000	[19]
	Atlantic Ocean	Portugal	<0.02–178	[31]
	Black Sea	Romania	5.50–69.6	This study
Caffeine	Mediterranean Sea	Spain	327	[32]
	Red Sea	Saudi Arabi	7708	[30]
	Black Sea	Romania	7.90–13,575	This study

**Table 9:** The environmental risk assessment of pharmaceuticals determined in seawater samples.

Compound	Toxicity	Species	End Point	µg/L	Reference	MEC µg/L	AF	PNEC	RQ	Level Risk
DIC	Chronic	*Daphnia magna*	LC50	2000	[44]	0.063	100	20	0.003	Very low
	Acute	*Lemna minor*	EC50	7500	[45]	0.063	1000	7.5	0.008	Very low
	Chronic	*Oncorhynchus mykiss*	LOEC	1	[46]	0.063	100	0.01	6.3	
IBU	Chronic	*Daphnia magna*	LC50	3970	[44]	0.134	100	39.7	0.003	Very low
	Chronic	*Parocentrotus lividus*	EC50	0.01	[47]	0.134	100	0.0001	1340	High
	Chronic	*Carcinus maenas*	LOEC	5	[48]	0.134	100	0.05	2.68	High
NAP	Chronic	*Thamnocephalus platyurus*	EC50	560	[49]	0.07	100	5.6	0.012	Low
	Chronic	*Ceriodaphnia dubia*	EC50	330	[49]	0.07	100	3.3	0.021	Low
CAF	Acute	*Ceriodaphnia dubia*	LC50	60,000	[50]	13,575	1000	60	0.226	Medium
	Acute	Fathead minnow (*Pimephales promelas*)	LC50	10,000	[50]	13,575	1000	10	1.35	High
	Acute	*Daphnia magna*	NOEC	5.2	[51]	13,575	1000	0.005	2611	High
KET	Acute	*Danio rerio*	LC50	1520	[52]	0.014	1000	1.52	0.009	Very low
	Acute	*Ceriodaphnia silvestrii*	EC50	24,840	[53]	0.014	1000	24.84	0.0006	Very low
	Acute	*Pseudokirchneriella subcapitata*	EC50	240	[54]	0.014	1000	0.24	0.058	Low

## Data Availability

The original contributions presented in this study are included in the article. Further inquiries can be directed to the corresponding authors.
